# The D-linking effect on extraction from islands and non-islands

**DOI:** 10.3389/fpsyg.2014.01493

**Published:** 2015-01-05

**Authors:** Grant Goodall

**Affiliations:** Department of Linguistics, University of CaliforniaSan Diego, La Jolla, CA, USA

**Keywords:** filler-gap dependencies, D-linking, island constraints, working memory, sentence acceptability

## Abstract

“D-linked” *wh*-phrases such as *which car* are known to increase the acceptability of sentences with island violations. One influential account of this attributes the effect to working memory: the D-linked filler is easier to retrieve at the site of the gap and this leads to the amelioration in acceptability. Such an account predicts that this effect should occur in general with non-trivial *wh*-dependencies, not just in island environments. An experiment is presented here to test this prediction. *Wh*-questions with both D-linked and bare *wh*-phrases and with both island and non-island embedded clauses are presented to participants, who rate their acceptability on a 7-point scale. Results show that D-linking significantly increases acceptability in both island and non-island environments, in accord with analyses that attribute the effect to working memory. In addition, the increase in acceptability is uniform in both types of environments, suggesting that the island effect itself may not be attributable to working memory.

## Introduction

The contrast between *wh*-phrases such as *which car* in (1a) and *what* in (1b) has been a major topic of research over the last few decades (e.g., Pesetsky, 1987; Cinque, 1990; Szabolcsi and Zwarts, 1993).

(1) a. **Which car** did you buy?
    b. **What** did you buy?

Following terminology introduced in Pesetsky (1987), *wh*-phrases like *which car* are “discourse-linked or “D-linked,” in that they naturally prompt an answer chosen from referents already existing in the discourse, whereas *wh*-phrases like *what* do not. (1a), for instance, is typically taken to be asking about a set of cars already known to the speaker and hearer, while (1b), under its most natural reading, is not (see also Katz and Postal, 1964 and Kuroda, 1968).

This distinction has been claimed to have two major consequences for the syntax of *wh*-dependencies. The first has to do with clauses containing two or more *wh*-phrases. English requires that one of these appear at the left edge of the clause, and generally, the syntactically more prominent *wh*-phrase (e.g., the subject vis-à-vis an object) is strongly preferred to play this role, as in (2a) and (2b), even though the less prominent *wh*-phrase is able to when there is no other, as in (2c).

(2) a. I wonder **who** bought **what**.
    b. ^*^I wonder **what who** bought.
    c. I wonder **what** the man bought.

This is known as the Superiority effect (Chomsky, 1973). D-linking of the *wh*-phrases is claimed to weaken or erase this effect, such that any *wh*-phrase may appear at the left edge of the clause, as in (3) (Karttunen, 1977; Pesetsky, 1987; Comorovski, 1989).

(3) a. I wonder **which man** bought **which car**.
    b. I wonder **which car which man** bought.

The second major consequence has to do with the gaps that are obligatorily associated with *wh*-phrase fillers. These gaps are not permitted in certain environments within the clause, a phenomenon known as an island effect (Ross, 1967). (4a) and (4b) show two such island environments, while (4c) shows a non-island environment, in which a gap is permitted.

(4) a. ^*^What do you wonder [who bought __] ?
    b. ^*^What do you believe [the claim that the man bought __] ?
    c. **What** do you think [that the man bought __] ?

As with Superiority, island effects are claimed to be weakened or erased when the *wh*-phrase is D-linked (Maling and Zaenen, 1982; Cinque, 1990; Rizzi, 1990; de Swart, 1992; Kiss, 1993; Chung, 1994):

(5) a. **Which car** do you wonder [who bought __] ?
    b. **Which car** do you believe [the claim that the man bought __] ?

The above two consequences are surprising, at least initially, in that one might not expect *wh*-dependencies, which are often taken to be a quintessentially syntactic phenomenon, to be so sensitive to discourse-related factors. The effects of D-linking thus present an interesting puzzle, and a number of analyses have been proposed to explain them.

This paper explores this second consequence, the effect of D-linking on islands. We present evidence from a formal acceptability experiment showing that D-linking does indeed improve acceptability of sentences containing island violations, but that they are still significantly degraded compared to sentences without such violations. Moreover, D-linking results in a similar improvement in acceptability even in non-island environments, a finding that has important consequences for determining the sources of the D-linking and island effects.

We review the main classes of proposed explanations for the D-linking effect in islands in Section Three Accounts of D-linking and consider earlier acceptability experiments in this domain in Section Earlier Acceptability Studies. Section Experiment presents and discusses the experiment itself. Section Implications for Formal Acceptability Experiments discusses implications of the experiment for acceptability experiments in general, and general conclusions are presented in Section Conclusion.

## Three accounts of D-linking

One influential analysis (Szabolcsi and Zwarts, 1993, 1997; see also Honcoop, 1998) claims that the D-linking effect in islands is primarily due to semantic factors. Certain island domains, under this analysis, contain operators that require a Boolean operation (e.g., intersection), which in turn requires sets made up of discrete individuals. A D-linked *wh*-phrase facilitates an interpretation in which the set questioned consists of individuals, thus allowing for a coherent semantic interpretation of the sentence. With bare *wh*-words like *what*, on the other hand, an interpretation involving a set of individuals is unlikely (though possible under certain circumstances, as Szabolcsi and Zwarts discuss), so the sentence is perceived as ill-formed.

In another set of analyses, the source of the unacceptability of island violations such as (4a) is syntactic. In Rizzi (2001, 2004), for instance, the *wh*-dependency between *what* and its gap site in (4a) violates a putative fundamental property of syntax known as Relativized Minimality, which roughly speaking, disallows dependencies between a filler and a gap when there is an intervening filler [*who*, in the case of (4a)] that could also potentially enter into a dependency of the same type with this gap. Fronted topics are known to be immune to Relativized Minimality effects, so it is important to note in this analysis that D-linked *wh*-phrases bear certain crucial similarities to fronted topics: they contain lexical material beyond the *wh*-word itself, and they are dependent on previously mentioned elements in the discourse. To the extent that D-linked *wh*-phrases may be interpreted as topics, then, they should be able to circumvent the Relativized Minimality requirement and acceptability should increase.

In a third family of analyses, island violations such as (4a) result from limitations in working memory (Kluender and Kutas, 1993; Kluender, 1998; Hofmeister and Sag, 2010; Hofmeister, 2011). The filler *what* must be held in working memory until it can be reintegrated into the structure at the gap site in the embedded clause. Maintaining this filler in working memory while also processing a clause boundary and an intervening filler (*who*) overwhelms the limited capacity of the processor, so filler reintegration is less likely to succeed and the sentence is perceived as unacceptable. The situation changes when the filler is D-linked, because such a filler requires more initial processing, given its more referential nature and the presence of lexical material. The D-linked filler thus has a higher level of initial activation in working memory, and this enables it to survive more successfully until the point where it can be reintegrated at the gap site. There is considerable evidence that such a processing advantage for D-linked fillers exists (e.g., Kluender, 1998; Frazier and Clifton, 2002; Diaconescu and Goodluck, 2004; Hofmeister, 2007a,b, 2011; Hofmeister and Sag, 2010; Hofmeister et al., 2013), and it is reasonable to assume that it could result in higher acceptability [see Hofmeister et al., 2007, for an application of this type of analysis to the D-linking effect on Superiority, as in (2)-(3) above].

This working memory account of islands and D-linking differs from the other two in two important ways. First, it claims that the island and D-linking effects are essentially extragrammatical. That is, the grammar itself has nothing to say about island structures and D-linked fillers, other than that they are allowed, and the effects observed result from capacity constraints on working memory. In the other accounts, on the other hand, these same effects arise because the sentences in question would require an ill-formed semantic or syntactic structure, independently of how such a structure would be processed. Second, all three accounts attribute special properties to D-linked fillers, but only in the working memory account would these special properties be expected to increase acceptability even without an island structure. More concretely, D-linked fillers more readily allow for individuation in the semantic account and for a topic-like interpretation in the syntactic account. These properties permit the filler to avoid island effects, but there is no reason to expect them to affect acceptability in non-island environments. In the working memory account, however, the special property of D-linked fillers is that they have a higher level of activation, and this should facilitate retention in working memory and reintegration at the gap site regardless of the particular structure. Since easier reintegration is assumed to result in higher acceptability, this then predicts that making a filler D-linked will increase acceptability in both island and non-island environments.

We thus arrive at a clear distinction between the working memory analysis and the other two: The working memory account predicts that D-linking will increase acceptability in both islands and non-islands, while the grammatical (semantic and syntactic) accounts do not make this prediction. On the other hand, the three analyses are in agreement that without any auxiliary assumptions, whatever D-linking effect occurs in non-islands should be smaller than that in islands. In the grammatical accounts, this is straightforward: no prediction is made for non-islands, but a very clear effect is predicted for islands. In the working memory account, the predictions result from the way in which island phenomena themselves are accounted for. In these analyses, islands occur because of two main factors: the processing difficulty associated with a filler-gap dependency and that associated with a particularly complex embedded clause [such as the *wh*-clause in (4a) or the complex noun phrase in (4b)]. Crucially, there is an interaction between these two factors, in that the decline in acceptability when both occur together is greater than what would be expected given the decline associated with each one on its own. Assuming that this interaction is straightforward (e.g., multiplicative), a weakening of one of the factors by amount *x* should result in an overall effect greater than *x*. More specifically, if D-linking lessens the processing difficulty found with filler-gap dependencies, the effect should be amplified when this difficulty is in interaction with the difficulty stemming from a complex embedded clause, and we thus expect D-linking to have a greater effect on acceptability in islands than it does in non-islands.

Two questions may now be posed: (i) Does D-linking increase acceptability in both islands and non-islands, and (ii) is the effect larger in islands than in non-islands? If the answer to the first question is positive, this would lend support to the working memory account of D-linking, and if it is negative, this would argue against it. As we have seen, the grammatical accounts do not make a specific prediction with regard to this question. As for the second question, a positive answer would confirm the predictions made by both the working memory and grammatical accounts. A negative answer would be consistent with the working memory account of D-linking, though inconsistent with the working memory account of islands, given straightforward assumptions about the nature of the interaction taken to underlie island effects. With regard to grammatical accounts, on the other hand, a negative answer would be inconsistent with the accounts of D-linking, though consistent with accounts of islands.

## Earlier acceptability studies

The questions that we are now facing, whether D-linking of fillers increases acceptability even in non-island environments and whether the effect is greater in islands than in non-islands, are in principle able to be addressed experimentally, and some earlier studies have attempted to do so. Hofmeister (2007a) reports the results of a pilot study exploring the effect in non-islands, in which 16 subjects rate 9 sentences using a 7-point scale. The fillers are bare *wh*-words or phrases consisting of either *which* + noun or *which* + *of* + *the* + noun, as in the sample stimuli in (6).

(6) a. Justin proved **what** the engineers lied that they had invented __ without any help or instruction.
    b. Justin proved **which devices** the engineers lied that they had invented __ without any help or instruction.
    c. Justin proved **which of the devices** the engineers lied that they had invented __ without any help or instruction.

The differences in acceptability among the sentences are marginally significant, with type (6b) more acceptable than (6c), and (6c) more than (6a), but given the small-scale nature of the experiment and the lack of clear results, it is difficult to draw firm conclusions from this. Nonetheless, the study shows that designing an experiment that begins to address these questions is possible in principle.

Alexopoulou and Keller (2013) report on a study consisting of two sub-experiments. In one, the stimuli consist of *wh*-questions with gaps inside embedded *whether*-clauses, a known island environment. In the other, the gap is either in the main clause or in an embedded *that*-clause. In both sub-experiments, there are two factors: gap type (true gap vs. resumptive pronoun) and filler type (*what* vs. *what* + noun vs. *which* + noun vs. *which* + *of* + *the* + noun). Samples of the stimuli with a gap in a *whether*-clause are given in (7a), in the main clause in (7b), and in a *that*-clause in (7c).

(7) a. **What**/**What movie**/**Which movie**/**Which of the movies** does Jean wonder [whether they will watch __ at the cinema]?
    b. **What**/**What movie**/**Which movie**/**Which of the movies** will they watch __ at the cinema?
    c. **What**/**What movie**/**Which movie**/**Which of the movies** does Mary think [they will watch __ at the cinema]?

The stimuli are arranged in 8 lists using a Latin square design, and subjects respond to the stimuli using magnitude estimation (Bard et al., 1996).

Alexopoulou and Keller find some evidence of a D-linking effect in the *whether*-island case, with *which* + noun (though not *what* + noun or *which* + *of* + *the* + noun) resulting in significantly higher acceptability than bare *what* in cases like (7a). Crucially, however, this effect is not found in either of the non-island environments (see Sprouse et al., for a similar finding, though with D-linked vs. bare as a between-subjects factor). That is, when the gap is in the matrix clause, as in (7b), or in an embedded *that*-clause, as in (7c), there is no significant difference among the four filler types. As discussed above, a result such as this presents straightforward evidence against the working memory account of the D-linking effect, since this account predicts that D-linked fillers will be easier to reintegrate into the structure and that this will lead to increased acceptability, both in island and non-island contexts. The lack of an observed effect in the non-island contexts is entirely consistent with the grammatical accounts and thus provides an argument in their favor.

(7b-c) are standardly considered fully acceptable with any of the fillers, however, so in order to detect a D-linking effect in these cases, the experiment will need to be able to distinguish among sentences at the very high end of the acceptability scale. There is some indication in Alexopoulou and Keller's results that their experiment is not able to do this reliably. Sentences with short dependencies as in (7b), where the filler and the gap are within the same clause, have always been found in previous experimental work to be much more acceptable than those with long dependencies, such as in (7c), where the filler and the gap are in separate clauses, despite the fact that both are standardly treated as grammatical (e.g., Cowart, 1997; Alexopoulou and Keller, 2007). In Alexopoulou and Keller's results, though, the two sentence types are virtually identical, strongly suggesting the presence of a ceiling effect. If this is true for short vs. long filler-gap dependencies, for which the literature reports a very robust difference, then the fact that they find no difference among the four filler types is perhaps not as telling as it appears at first.

A similar lack of expected distinctions in the mid-range of the acceptability scale suggests that the experiment may not have attained a level of sensitivity sufficient to detect all potential contrasts of interest. The *whether*-islands tested are a canonical example of the type of island that is thought to exhibit D-linking effects (see, e.g., Szabolcsi, 2006), yet recall that this was only found with *which* + noun, not *what* + noun or *which* + *of* + *the* + noun, contrary to expectations. The absence of a D-linking effect with *that*-clauses in this experiment is thus perhaps not surprising, given that this effect was also not detected in some of the cases where it would be most expected.

The possibility that the experiment was not sensitive enough to detect all potential D-linking effects gains further plausibility when one looks at the details of the experimental design, which show several features that could have contributed to a lowered level of sensitivity. In terms of the materials, each participant saw just one token of each condition, and there was a 1:1 filler/experimental ratio. In addition, there was only partial counterbalancing of the stimuli: There were 24 conditions overall, yet only 8 lexicalizations of each condition, and 8 lists of stimuli were created. These lists were distributed among 22 participants, so some lists (and stimuli) were seen by more participants than others. As for the participants themselves, they were self-reported native speakers of English recruited over the Internet. Given the nature of the English-speaking community, where bilingualism in many forms is very common and it is not always clear who counts as a “native speaker,” it is possible that the participants' language histories were very heterogeneous, which in turn could have led to increased variability in their responses. In addition, participants took part in the experiment over the Internet. Although indications are that performing sentence acceptability experiments in this way gives adequate results (Gibson et al., 2011; Sprouse, 2011b), there is still the realistic possibility that it will result in increased noise, especially when the number of participants is small. Finally, the response method used with participants (magnitude estimation) may have also contributed to a decrease in sensitivity. This is still a matter of some controversy, but there are suggestions in the literature that magnitude estimation may not be as sensitive as initially thought and that it may even obscure fine-grained distinctions (Sprouse, 2011a; Weskott and Fanselow, 2011; Fukuda et al., 2012).

We of course cannot be sure that any of the above factors resulted in a decrease in the experiment's sensitivity, but given that the D-linking effect is likely very subtle, it would be prudent to avoid design features that might make detecting such an effect more difficult.

Given the existing literature, then, it is still an open question whether D-linking increases the acceptability of *wh*-dependencies in non-island environments and if it does, whether this effect is smaller in non-islands than in islands. In the Hofmeister (2007a) study, the results are not clear enough to draw firm conclusions, and in the Alexopoulou and Keller (2013) study, there are reasons to suspect that the results are compromised by a ceiling effect and a general lack of sensitivity. In the following section, we describe an experiment that is designed to address directly the questions of a possible D-linking effect in non-island environments and how this might compare to that in island environments.

## Experiment

### Participants

Fifty six people participated in this experiment. All were undergraduate students at the University of California, San Diego who were participating for course credit. The experiment was performed in a laboratory setting, with prior authorization from the university's Institutional Review Board. All participants gave their informed consent.

The results of two groups of participants were excluded. The first included those who on a language background questionnaire, gave a language other than English as their native language or their dominant language, or who indicated that they had been born outside of the U.S. This eliminated 6 participants. The second group included those who did not appear to be attending to the task, as evidenced by their responses on 9 key filler items that were unquestionably grammatical or unquestionably ungrammatical. Participants who made 2 or more “errors” on these fillers were excluded, where “errors” are defined as a response of 3 or below (on a 1–7 scale) to a grammatical filler or a response of 5 or above to an ungrammatical filler. 2 participants were eliminated in this way, leaving 48 in total (2 per experimental list).

### Materials and method

Experimental items were all *wh*-questions and were prepared using a 2 × 3 design, crossing filler type (bare vs. D-linked) and type of structure in which the gap is located (embedded complex noun phrase vs. *wh*-clause vs. *that*-clause). With regard to filler type, the bare filler was always *what* and the D-linked fillers all had the form *which of the* + plural noun. With regard to structure type, the complex noun phrases all contained a singular head noun (e.g., *claim, plan, idea*), followed by a clausal complement, and the *wh*-clauses all contained *who* as subject of that clause. The 6 conditions are exemplified in (8).

(8) a. **What** / **Which of the cars** do you believe the claim that he might buy?
    b. **What** / **Which of the cars** do you wonder who might buy?
    c. **What** / **Which of the cars** do you believe that he might buy?

(8a) and (8b) are classic violations of island constraints: the Complex Noun Phrase Constraint (CNPC) and the *Wh*-island Constraint, respectively (Ross, 1967). The gap in (8c) is within a *that*-clause, a classic non-island environment.

Twenty four sets of lexically matched stimuli were created and distributed into 6 counterbalanced lists using a Latin square design, such that each list contained 4 tokens of each condition. 81 filler items were added to each list, and the lists were then pseudo-randomized twice, resulting in 12 lists. An additional 12 lists were created by reversing the order of items, resulting in a total of 24 lists. 2 participants were randomly assigned to each list; each experimental item was thus seen by 8 participants. The full set of stimuli is presented in the Supplementary Material.

Participants saw the stimuli on a computer screen and were instructed to rate each sentence on a scale from 1 (“very bad”) to 7 (“very good”) based on how it sounded to them as a native speaker of the language. The scale was presented horizontally in evenly spaced increments with only the two extremes labeled and participants indicated their response by clicking on the appropriate number. They were told to rely on their first reaction, without trying to analyze the sentence, and that there were no “correct” answers. They were also told to rate each sentence on its own, regardless of how simple or complicated the sentence might seem.

### Results

**Figure 1:**
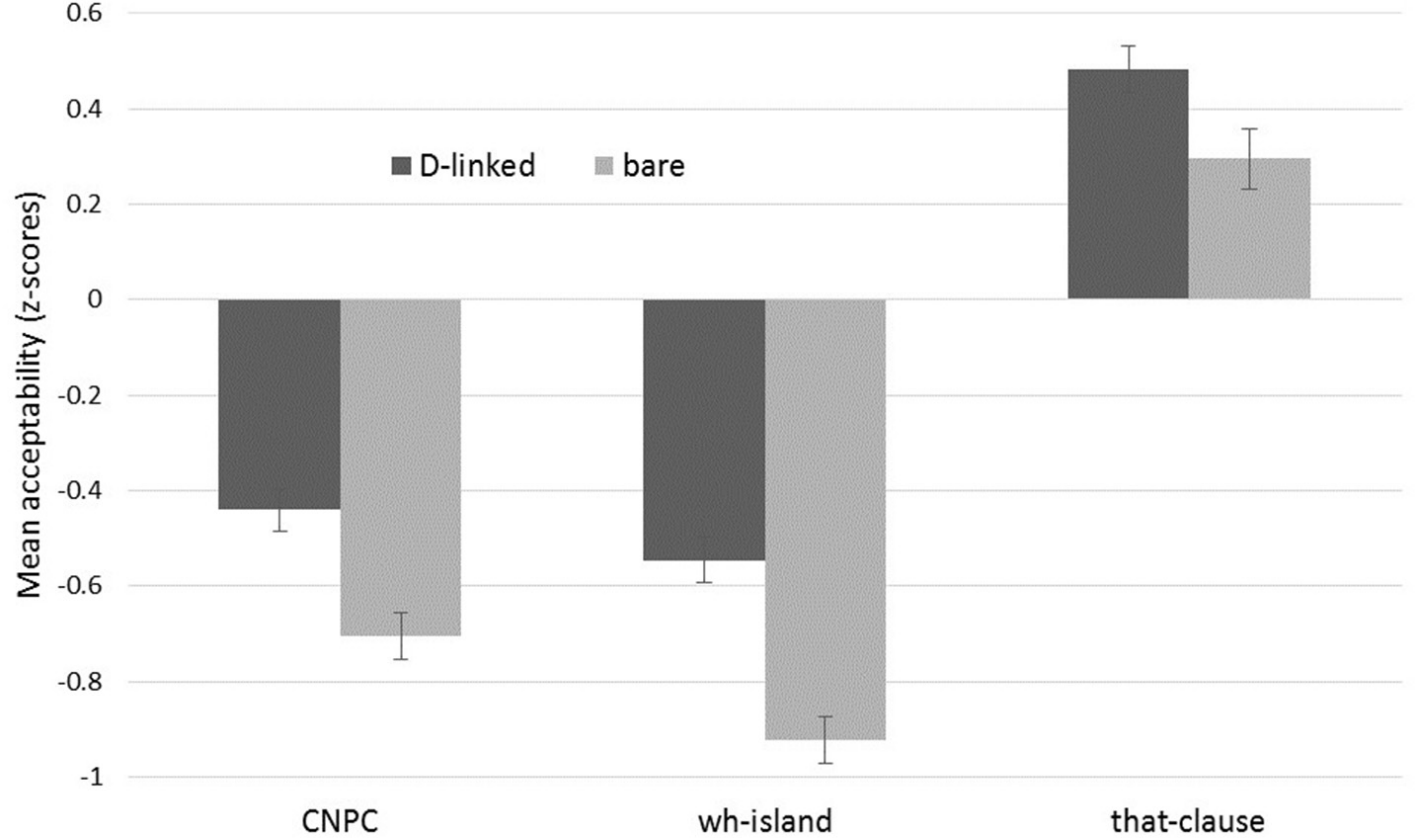
**Mean acceptability of experimental conditions (z-scores; error bars indicate SE)**.

The results were transformed to z-scores prior to analysis. The z-score mean and standard error for each of the six conditions is presented in Figure 1.

A linear mixed effects model was run with filler type and structure type as fixed factors, participant and item as random intercepts, and by-participant and by-item random slopes for filler type and a by-participant random slope for structure type, using the *lmer* function in the *lme4* package for R (Bates et al., 2014a,b; R Core Team, 2014). All *p*-values were calculated by Satterthwaite approximation, using the *lmerTest* package (Kuznetsova et al., 2014). This revealed a significant main effect for filler type (D-linked: −0.168 vs. bare: −0.444; *t* = 3.446; *p* < 0.001), and this effect remained significant when the model was restricted to each of the three structures individually: CNPC (D-linked: −0.441 vs. bare: −0.705; *t* = 3.476; *p* < 0.01), *wh*-island (D-linked: −0.545 vs. bare: −0.923; *t* = 3.982; *p* < 0.001), and *that*-clause (D-linked: 0.483 vs. bare: 0.295; *t* = 2.416; *p* < 0.02). To test for an interaction between filler type and structure type, a second model was constructed without an interaction between these two fixed factors and the results compared to the first by means of the *anova* function. This revealed no significant difference between the two models (*p* = 0.155) and thus no significant interaction between these two factors. The interaction between filler type and structure type was also not significant when the CNPC data were excluded and the model run as a 2 × 2 design, with *wh*-island and *that*-clause as the levels for structure type (*t* = 1.866; *p* = 0.062) and when the *wh*-island data were excluded and CNPC and *that*-clause used as the levels for structure type (*t* = 0.771; *p* = 0.440).

To a large extent, earlier observations in this domain are confirmed (e.g., general island effects and D-linking effects are readily apparent), but there are two novel findings here. First, the increase in acceptability associated with D-linked fillers occurs in all three structure types, not just in the islands. Second, this increase is uniform across all three types. That is, the amount of increase associated with D-linking does not appear to vary significantly between islands and non-islands.

**Figure 2:**
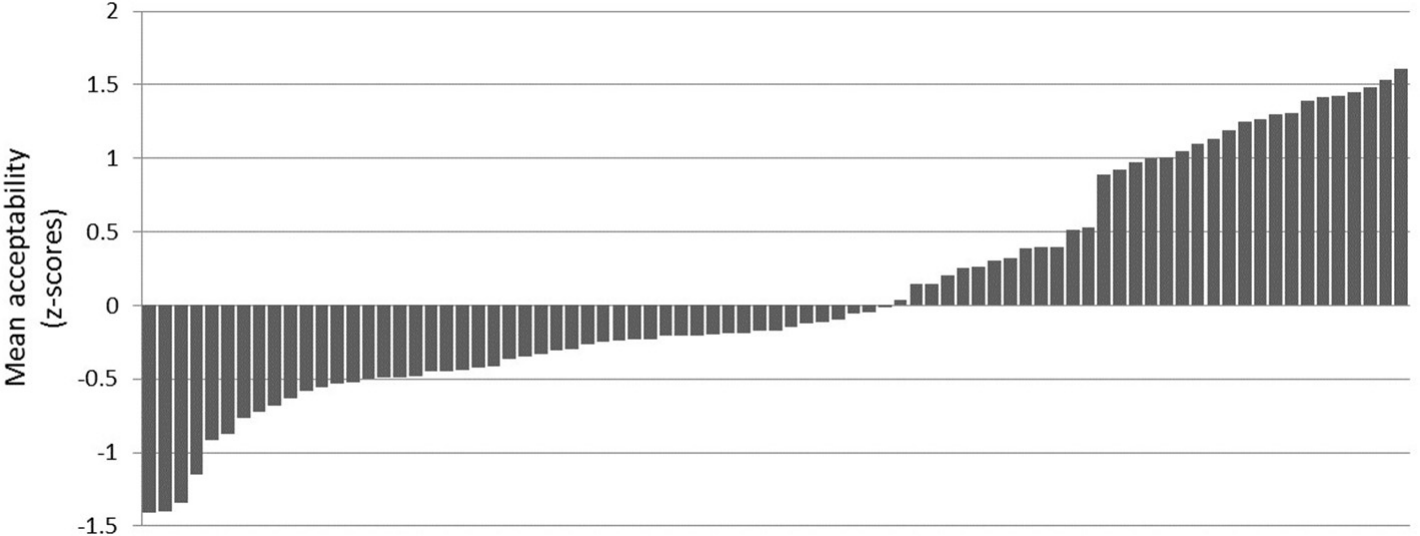
**Mean acceptability of fillers (z-scores)**.

As noted earlier, achieving sufficient sensitivity is a concern in this type of study, but the fact that all of the island effects and D-linking effects that the existing literature predicts did emerge suggests that the experiment was successful in this regard. It also appears that the experiment avoided a ceiling effect in the case of *wh*-questions with a gap in the *that*-clause. Although significantly more acceptable than the island violations, these sentences are still within the mid-range of the acceptability of the fillers. As seen in Figure 2, the acceptability of the fillers went as high as 1.61, much higher than the mean acceptability of the *that*-clause sentences with either a bare or D-linked filler (0.295 and 0.483, respectively).

### Discussion

The main purpose of this study is to determine whether D-linking of the filler improves the acceptability of *wh*-questions where the gap is in a non-island, and if so, whether this improvement is of the same size as that which occurs when the gap is within an island. We have now seen that the effect does occur in non-islands and that it is not different in size from that observed in islands. More specifically, D-linking leads to a significant increase in acceptability when the gap is in a non-island *that*-clause, and in addition, significant increases are also found in the two island cases examined. There is no significant interaction between filler type and structure type, suggesting that the amelioration due to D-linking is essentially uniform regardless of whether the gap is within an island or non-island.

These results confirm one crucial prediction of the working memory analysis of D-linking effects. If, as this analysis claims, D-linking effects arise because the nature of D-linking allows for easier reintegration of the filler at the gap site, and if this in turn results in higher acceptability, then we would expect to be able to detect this increase in acceptability no matter whether the gap is located in an island or a non-island. The results seen here suggest that this prediction is correct and thus provide new evidence in favor of the working memory analysis. This new evidence from acceptability complements and is in accord with the considerable evidence already existing that D-linking facilitates the processing of filler-gap dependencies.

The results of the experiment are at odds, however, with another prediction that is shared by both the working memory analysis and the grammatical analyses. Namely, the experiment finds an essentially uniform D-linking effect in both islands and non-islands, whereas both types of analyses, in their most straightforward forms, predict a larger effect in the case of islands. For the working memory analysis, this is because island phenomena are the result of an interaction between the difficulty of the dependency and the difficulty of the structure, so if we assume that this interaction is simple (e.g., multiplicative), facilitating the dependency in this case should lead to an increase in the acceptability of the island that is larger than what would be expected by facilitating the dependency alone, as in a non-island structure. For the grammatical analyses, it is because island phenomena are the result of limitations on the operation of the syntax and/or semantics, and D-linking has the effect of removing these limitations. In non-islands, these limitations do not exist, so no effect of D-linking is expected. Both the working memory and the grammatical analyses, then, predict a difference in behavior between islands and non-islands with regard to D-linking, but this difference is not found here.

On the one hand, then, the results of the experiment here provide important support for the idea that the D-linking effect is ultimately due to an effect of working memory. We have found that D-linking increases acceptability in both island and non-island environments, just as would be expected if D-linking facilitates reintegration of the filler at the gap site in filler-gap dependencies. On the other hand, though, the results suggest caution with the idea that the island phenomenon itself is ultimately due to working memory. As we have seen, we would expect a larger D-linking effect in islands than in non-islands if this were true, and this is not what we observe. The results here are most compatible, then, with the view that the D-linking effect is due to working memory and that the island effect is due to some independent mechanism. Crucially, this mechanism and the working memory effect should be such that they do not interact, as would be expected, for example, if the island effect (but not the D-linking effect) were the result of a grammatical constraint. Given the types of grammatical constraints that have been proposed for islands (e.g., Rizzi, 2004; Boeckx, 2008; Truswell, 2011), one would expect them to combine additively with working memory effects, without any interaction, and the results here thus provide some support for such an account of islands and D-linking. Clearly, though, any conclusion that islands themselves are independent of working memory effects must be approached with caution, given the evidence that has been put forward suggesting that the two are closely related (for recent discussion of the evidence for and against this idea, see Hofmeister et al., 2012a,b; Sprouse et al., 2012a,b; and Michel, 2014).

Further support for the idea that the D-linking effect itself is due to the effects of working memory comes from an experimental result not yet highlighted: both CNPC and *wh*-islands show a significant amelioration with D-linking. This finding is of interest because much of the literature on D-linking assumes that it affects only *weak* islands (i.e., those in which acceptability of an argument gap is much higher than that of an adjunct gap) and not *strong* islands (i.e., those in which argument gaps and adjunct gaps are equally unacceptable) (e.g., Cinque, 1990). *Wh*-islands are a standard example of a weak island and CNPC is typically taken to be a strong island (e.g., Szabolcsi, 2006), so the fact that both show a clear D-linking effect in the results here runs counter to common assumptions. It is exactly what the working memory analysis of D-linking predicts, however, so this finding represents additional support for it.

Another area where the experimental results here run counter to common assumptions in the literature concerns the relation between D-linking and islands. It is often stated that D-linking makes gaps within islands licit (e.g., Szabolcsi, 2006). The results here point to a more nuanced view, however. Although a D-linked filler does significantly increase the acceptability of a gap within an island, this increased acceptability is still relatively low: the mean z-scores are well below 0 (−0.441 for CNPC and −0.545 for *wh*-islands) and below most of the filler items (see also Goodall, 2004, 2010; and Sprouse et al., in press). The contribution of D-linking to acceptability seen here may thus be more modest than what is sometimes suggested, but this fact is compatible with both processing and grammatical analyses of D-linking. In the processing analyses, the idea that D-linking leads to easier reintegration of the filler at the gap site does not mean that no difficulty remains, and this residual difficulty would reasonably be expected to lead to low acceptability. In the grammatical analyses, similarly, D-linking may make it easier to construe the filler as being individuated or as referring to material in the previous discourse, but it is very conceivable that such accommodation would come with a processing cost that would suppress acceptability. The fact that the increase in acceptability due to D-linking is relatively small is thus important to note, but it does not in itself necessarily differentiate among various analyses of the D-linking effect.

The experiment here was designed to test for D-linking effects across a range of syntactic environments. As is always the case, one must be cautious about generalizing the results beyond those structures tested. The experimental design included reasonable representative samples of a non-island structure (*that*-clause), for instance, and of island structures (CNPC and *wh*-islands), but these of course do not exhaust the possibilities (see Sprouse et al., in press, for an investigation of subject and adjunct islands, in addition to those explored here). Similarly, the type of D-linked filler used (*which of the* N) is a prototypical one, but there are other possibilities (*which* N or *what* N) that could also be tested. In addition, the stimuli in this experiment were presented without context (although by their very nature, D-linked fillers provide a kind of context that bare fillers do not), but D-linking is known to be sensitive to context, to such an extent that even bare *wh*-words can behave as D-linked *wh*-phrases if the context is strong enough (e.g., Cinque, 1990; Szabolcsi and Zwarts, 1993; Rizzi, 2004). There is no particular reason to expect that manipulating either the island/non-island structure or the properties of the filler would alter the results presented here, but prudence dictates caution in extending too far beyond what this study provides evidence for.

## Implications for formal acceptability experiments

The use of formal experiments to measure acceptability is relatively recent, primarily coming after the publication of Schutze (1996) and Cowart (1997), and has only become common in the last few years (see, e.g., Myers, 2009 and Sprouse and Hornstein, 2013 for overviews). As a consequence, there are still certain methodological concerns and questions for which there does not yet exist a full consensus, and some of these relate to aspects of the present study.

**Figure 3:**
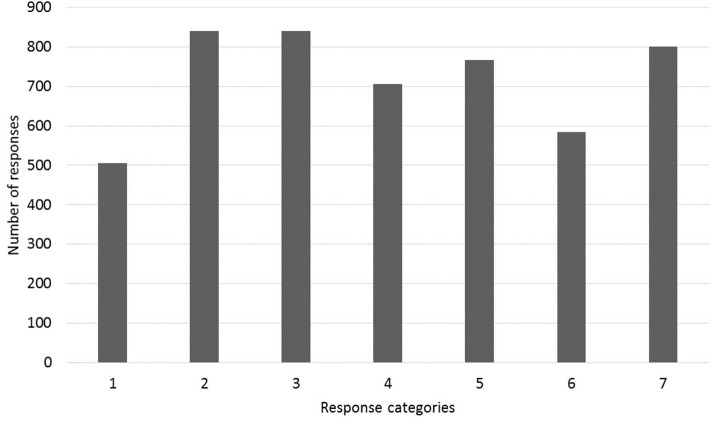
**Distribution of the 5040 participant responses per response category**.

One of these concerns the proper way to interpret participant responses to stimuli on the numerical scale. In this study, as in many others, participants were asked to indicate their responses using a 7-point scale, where 1 was labeled “very bad” and 7 “very good.” This method is known to yield results that are reasonably valid, reliable and sensitive (Myers, 2009; Weskott and Fanselow, 2011; Fukuda et al., 2012), but there remain concerns that participants may use different areas of the scale in different manners. In particular, Poulton (1979, 1989) demonstrates equalizing biases in rating tasks in which participants spread out responses over the full range of the scale and tend to use each response category equally often. In acceptability studies, this means that if there were a large number of low-acceptability stimuli and many fewer high-acceptability stimuli, for example, the differences among the lower ones could be exaggerated (i.e., participants would spread their responses out over a larger portion of the scale) while differences among the higher ones could be suppressed (i.e., participants would compress their responses into whatever portion of the scale was not being used for the lower stimuli). A possibility like this is a special concern in the present study for two reasons. First, the essential question being asked is whether a small difference in the lower end of the scale (i.e., the D-linking effect in island environments) is also found in the higher end of the scale (i.e., in non-island environments). Since this latter difference was indeed found, one could legitimately worry that this finding results simply from a tiny difference being exaggerated because of an equalizing bias. Second, there is some initial indication that the results are consistent with an equalizing bias, in that many of the response categories were used at similar rates, as seen in Figure 3 (especially categories 2, 3, 5, and 7), and furthermore, the number of responses at the lower end (categories 1–3) and at the higher end (categories 5–7) of the scale were almost identical: 2183 and 2151, respectively.

**Figure 4:**
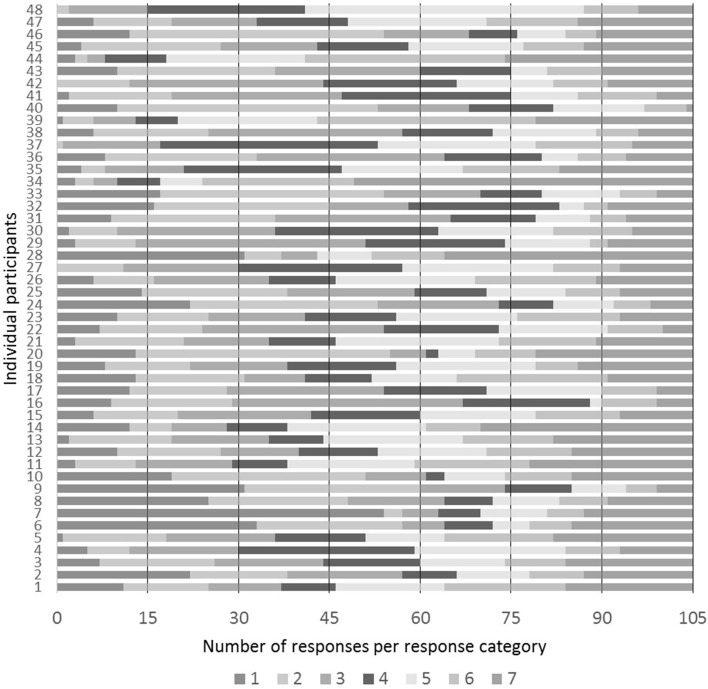
**Distribution of responses by individual participant**. An equal distribution of responses would consist of 15 responses per category per participant.

There is thus a real concern that the results are influenced by an equalizing bias on the part of participants. However, closer inspection of participant responses reveals that despite the overall distribution in Figure 3, most individual participants used the seven response categories at very uneven rates, as seen in Figure 4, suggesting that there was no clear equalizing bias for most participants.

Moreover, Cowart (1997), notes that rating experiments can be designed so as to discourage the possibility of equalizing bias. For example, the stimuli (including filler items) can be created so that no particular area of the scale is likely to predominate, thus decreasing the possibility of distortion in one area of the scale. In addition, the response scale can be presented to subjects in such a way that clearly invites an interpretation of the numbers as representing equal intervals. Both of these measures were taken in the present study. The stimuli included many filler items that were unquestionably of very high acceptability, as in (9), and of very low acceptability, as in (10), as well as many of intermediate status, as in (11).

(9) What do you think was on the table yesterday? (raw mean = 6.67).
Are all of the children in the room? (raw mean = 6.88).
(10) What would the girl could the tiger suddenly do? (raw mean = 1.54).
Would the this store is successful? (raw mean = 1.54).
(11) What does everybody say that Marge saw the books? (raw mean = 2.69).
Who were sculptures of on exhibit in the gallery? (raw mean = 3.58).
About which bike will several ads be shown to the athletes? (raw mean = 4.61).

Second, the response categories were presented after each stimulus in left-to-right increasing order in evenly spaced increments, in the manner of a ruler, with each numeral underneath its corresponding response button. Neither of these steps can eliminate the possibility of response biases, but together, they make it more likely that the D-linking amelioration that we observed with non-islands at the higher end of the scale is in fact similar and comparable to the amelioration seen with islands at the lower end of the scale.

Another area of concern in the recent literature on formal acceptability experiments has been cases where the experimental results and those obtained through more traditional means (i.e., by asking a small number of speakers (perhaps including the investigator) for judgments on a representative set of sentences) seem to diverge (Sprouse and Almeida, 2012, 2013; Gibson and Fedorenko, 2013; Gibson et al., 2013; Sprouse et al., 2013). The present experiment is of interest in this regard, because some of the results align with the traditional literature and others do not. For instance, the D-linking effect that was observed here with *wh*-islands lines up well with what has been reported in more traditional studies, but the similar effect seen with *that*-clauses does not. This then leads to a clear question: If there really is a D-linking effect with gaps in *that*-clauses, why has this never been observed in studies using more traditional methodology? Two possible answers arise. First, it may be simply that no one found this effect because no one was looking for it. From the standpoint of a researcher exploring properties of the grammar, gaps within *that*-clauses are highly acceptable and thus presumably grammatical (i.e., allowed by the grammar). Finding that these gaps become even more acceptable when the filler is D-linked would not be informative, because in standard models, there is no way for the sentence to become even more grammatical. Put simply, standard grammatical models can capture gradations of ungrammaticality (e.g., by counting the number of violations or their severity), but not gradations of grammaticality. From this standpoint, then, there would be no particular reason to look for D-linking effects in otherwise grammatical sentences.

A second answer might be that formal acceptability experiments appear to be very sensitive to strains on working memory in a way that more traditional methods are not, especially for sentences in the higher range of acceptability. For example, filler-gap dependencies within a single clause and those spanning two clauses are, other things being equal, taken to be equally acceptable in traditional studies, but formal acceptability experiments typically find a sharp decline in acceptability for the latter (Kluender and Kutas, 1993; Cowart, 1997; Alexopoulou and Keller, 2007). It is not clear why this divergence between the two methods occurs, but given that it does, the fact that the present study found a distinction that traditional studies have not begins to make sense. If the D-linking effect truly is a working memory effect, then we might not expect traditional methods to be sensitive to it in the case of *that*-clauses, which are of relatively high acceptability.

There thus appear to be reasonable ways in which one might explain the discrepancy between traditional methods and the experiment presented here with regard to the effect of D-linking in non-island environments. In this case or more generally, it is not a question of which of these methods is right or wrong, but of which is appropriate given the resources available and the nature of the phenomenon being investigated. Since the focus of investigation here concerns the possibility of small differences in acceptability among sentences that are taken to be grammatical, where working memory effects might crucially be involved, a formal experiment seems appropriate.

Finally, the present experiment highlights the fact that there is as much need for careful design and attention to detail in sentence acceptability experiments as in any other experimental methodology. Many of the acceptability contrasts that interest researchers are very robust and are easily detectable across a wide range of methodologies: traditional fieldwork, traditional introspection, very simple experiments, etc. For more subtle contrasts, however, the method may need to be chosen more carefully. In this study, several steps were taken in order to ensure adequate sensitivity and to avoid a ceiling effect, a particular danger in this case since the crucial sentences of interest were of relatively high acceptability. For example, participants were screened for language background and attention to task, and they performed the experiment in a laboratory setting. The materials were also fully counterbalanced: experimental stimuli were distributed across lists following a standard Latin square design, and each experimental item was seen by exactly the same number of participants. Filler items represented a wide range of acceptability, including many of very high acceptability. In addition, there was a relatively large number (192) of observations per condition (4 tokens of each condition per participant; 48 participants), and the response method used by participants (7-point scale) is one that has been shown capable of capturing small differences in acceptability (Weskott and Fanselow, 2011; Fukuda et al., 2012). These various aspects of the experimental design were chosen deliberately in response to the particular needs presented by this study.

## Conclusion

It has been known for many years that D-linking, where the filler in a *wh*-question prompts an answer chosen from referents already existing in the discourse, increases the acceptability of sentences where the gap is inside an island configuration. It has been claimed in a number of analyses that this phenomenon reflects the way that working memory operates in sentence processing, in that at the point of the gap site, D-linked fillers are easier to access and then integrate into the existing structure, and that this ease of processing results in higher acceptability. These analyses clearly predict that this D-linking effect should be found not just with islands, but with filler-gap dependencies in non-islands as well. The experiment presented here tested this prediction directly by probing for D-linking effects on acceptability in two island and one non-island environments. It was seen that the effect occurs in all three cases, confirming the prediction made by the analyses that attribute the effect to the operation of working memory.

In addition, the effect is essentially uniform across all three cases, contrary to what many analyses of the islands themselves would predict. The combined results are most compatible with a view in which the D-linking effect is due to working memory and the island effects are due to something independent of this, such as grammar. The results here suggest that these two effects may combine additively, but do not interact.

### Conflict of interest statement

The author declares that the research was conducted in the absence of any commercial or financial relationships that could be construed as a potential conflict of interest.
