# Supplementary material for: The D-linking effect on extraction from islands and non-islands
Source: Front Psychol. 2015 Jan 5;5:1493. doi: 10.3389/fpsyg.2014.01493 (PMC4283514; doi:10.3389/fpsyg.2014.01493)
Supplement: Supplementary file 1 [file DataSheet1.DOCX]

**Appendix: Stimuli**

Experimental items

Experimental stimuli were created with a 2 x 3 design, crossing filler type (bare vs. D-linked) and type of structure in which the gap is located (embedded complex noun phrase vs. *wh*-clause vs. *that*-clause), yielding 6 conditions. Each participant saw 4 items from each condition.

**Condition 1: Bare filler, CNPC**

What do you believe the claim that he might buy?

What do you accept the idea that she should read?

What do you approve of the fact that we will rent?

What do you reject the suggestion that he should study?

What do you like the plan that she will redesign?

What do you agree with the proposal that we should eliminate?

What do you reject the claim that he would sell?

What do you approve of the idea that she could adopt?

What do you accept the fact that we might chop down?

What do you like the suggestion that he should ride?

What do you agree with the plan that she will clean?

What do you believe the proposal that we should demolish?

What do you understand the claim that he could program?

What do you hate the idea that she might use?

What do you like the fact that we will visit?

What do you agree with the suggestion that he should photograph?

What do you approve of the plan that she should climb?

What do you reject the proposal that we will publish?

What do you doubt the claim that he might donate?

What do you like the idea that she could produce?

What do you understand the fact that we will invade?

What do you approve of the suggestion that he should wear?

What do you understand the plan that she could manage?

What do you accept the proposal that we will destroy?

**Condition 2: Bare filler, *wh*-clause**

What do you wonder who might buy?

What do you question who should read?

What do you need to know who will rent?

What do you wonder who should study?

What do you question who will redesign?

What do you need to know who should eliminate?

What do you wonder who would sell?

What do you question who could adopt?

What do you need to know who might chop down?

What do you wonder who should ride?

What do you question who will clean?

What do you need to know who should demolish?

What do you wonder who could program?

What do you question who might use?

What do you need to know who will visit?

What do you wonder who should photograph?

What do you question who should climb?

What do you need to know who will publish?

What do you wonder who might donate?

What do you question who could produce?

What do you need to know who will invade?

What do you wonder who should wear?

What do you question who could manage?

What do you need to know who will destroy?

**Condition 3: Bare filler, *that*-clause**

What do you believe that he might buy?

What do you think that she should read?

What do you suppose that we will rent?

What do you imagine that he should study?

What do you feel that she will redesign?

What do you assume that we should eliminate?

What do you sense that he would sell?

What do you understand that she could adopt?

What do you expect that we might chop down?

What do you know that he should ride?

What do you realize that she will clean?

What do you recognize that we should demolish?

What do you believe that he could program?

What do you think that she might use?

What do you suppose that we will visit?

What do you imagine that he should photograph?

What do you feel that she should climb?

What do you assume that we will publish?

What do you sense that he might donate?

What do you understand that she could produce?

What do you expect that we will invade?

What do you know that he should wear?

What do you realize that she could manage?

What do you recognize that we will destroy?

**Condition 4: D-linked filler, CNPC**

Which of the cars do you believe the claim that he might buy?

Which of the books do you accept the idea that she should read?

Which of the houses do you approve of the fact that we will rent?

Which of the planets do you reject the suggestion that he should study?

Which of the restaurants do you like the plan that she will redesign?

Which of the libraries do you agree with the proposal that we should eliminate?

Which of the hotels do you reject the claim that he would sell?

Which of the dogs do you approve of the idea that she could adopt?

Which of the trees do you accept the fact that we might chop down?

Which of the motorcycles do you like the suggestion that he should ride?

Which of the apartments do you agree with the plan that she will clean?

Which of the buildings do you believe the proposal that we should demolish?

Which of the computers do you understand the claim that he could program?

Which of the phones do you hate the idea that she might use?

Which of the villages do you like the fact that we will visit?

Which of the lakes do you agree with the suggestion that he should photograph?

Which of the mountains do you approve of the plan that she should climb?

Which of the books do you reject the proposal that we will publish?

Which of the paintings do you doubt the claim that he might donate?

Which of the movies do you like the idea that she could produce?

Which of the countries do you understand the fact that we will invade?

Which of the jackets do you approve of the suggestion that he should wear?

Which of the stores do you understand the plan that she could manage?

Which of the bridges do you accept the proposal that we will destroy?

**Condition 5: D-linked filler, *wh*-clause**

Which of the cars do you wonder who might buy?

Which of the books do you question who should read?

Which of the houses do you need to know who will rent?

Which of the planets do you wonder who should study?

Which of the restaurants do you question who will redesign?

Which of the libraries do you need to know who should eliminate?

Which of the hotels do you wonder who would sell?

Which of the dogs do you question who could adopt?

Which of the trees do you need to know who might chop down?

Which of the motorcycles do you wonder who should ride?

Which of the apartments do you question who will clean?

Which of the buildings do you need to know who should demolish?

Which of the computers do you wonder who could program?

Which of the phones do you question who might use?

Which of the villages do you need to know who will visit?

Which of the lakes do you wonder who should photograph?

Which of the mountains do you question who should climb?

Which of the books do you need to know who will publish?

Which of the paintings do you wonder who might donate?

Which of the movies do you question who could produce?

Which of the countries do you need to know who will invade?

Which of the jackets do you wonder who should wear?

Which of the stores do you question who could manage?

Which of the bridges do you need to know who will destroy?

**Condition 6: D-linked filler, *that*-clause**

Which of the cars do you believe that he might buy?

Which of the books do you think that she should read?

Which of the houses do you suppose that we will rent?

Which of the planets do you imagine that he should study?

Which of the restaurants do you feel that she will redesign?

Which of the libraries do you assume that we should eliminate?

Which of the hotels do you sense that he would sell?

Which of the dogs do you understand that she could adopt?

Which of the trees do you expect that we might chop down?

Which of the motorcycles do you know that he should ride?

Which of the apartments do you realize that she will clean?

Which of the buildings do you recognize that we should demolish?

Which of the computers do you believe that he could program?

Which of the phones do you think that she might use?

Which of the villages do you suppose that we will visit?

Which of the lakes do you imagine that he should photograph?

Which of the mountains do you feel that she should climb?

Which of the books do you assume that we will publish?

Which of the paintings do you sense that he might donate?

Which of the movies do you understand that she could produce?

Which of the countries do you expect that we will invade?

Which of the jackets do you know that he should wear?

Which of the stores do you realize that she could manage?

Which of the bridges do you recognize that we will destroy?

Filler items

Each participant saw 9 filler items from a fixed set and 72 items that were stimuli from two unrelated experiments that were administered concurrently as sub-experiments. These latter items were different for each participant, but a representative set is given here.

**Fixed filler items**

What would the girl could the tiger suddenly do?

What do you think was on the table yesterday?

What does everybody say that Marge saw the books?

Which advisors think that Bill should drive an electric car when he is in the city?

Would the this store is successful?

What will Tom buys when he could went on vacation?

Are all of the children in the room?

Should the students will studied on those bus?

Which teacher says that the course should contain material on the history of Ecuador in the colonial period?

**Filler items from other sub-experiments (representative set)**

Which animal do you wonder how many movies about will be shown to the visitors?

Which hobby do you know how many magazines about should be turned over to the librarians?

Which person did you ask how many secrets about would be divulged to the reporters?

Which machine did Jim find out how many brochures about will be mailed to the representatives?

Which airplane did Tim wonder whether several facts about could be revealed to the public?

Which airline did Michael find out whether several websites about were displayed to the passengers?

Which resort did Emily ask whether several brochures about will be brought to the conference?

Which technique did Chloe wonder whether several classes about should be suggested to the interns?

Which mistress could several letters about be revealed to the children?

Which assassination were several theories about explained to the students?

Which tree will several articles about be distributed to the gardeners?

Which city would several lectures about be provided to the tourists?

Which war do you know how many movies about they could present to the actors?

Which cathedral did Joan ask how many videos about they will award to the prizewinners?

Which topic does Katie wonder how many sessions about they should propose to the committee?

Which scandal does Emma know how many memos about they would disclose to the newspapers?

Which train do you wonder whether they donated several videos about to the children?

Which toothbrush do you know whether they will present several ads about to the dentists?

Which sport did Ava ask whether they should donate several magazines about to the hospital?

Which celebrity do you wonder whether they would disclose several secrets about to the fans?

Which disease should they recommend several sessions about to the organizers?

Which crisis would they give several memos about to the advisors?

Which country did they present several books about to the scholars?

Which politician could they offer several TV shows about to the networks?

About which proverb did Chris wonder how many sermons could be offered to the congregation?

About which athlete did Natalie find out how many documentaries were supplied to the teams?

About which dancer did Jacob ask how many films were recommended to the critics?

About which terrorist does Alex wonder how many programs will be sent to the investigators?

About which regulation did Courtney ask whether several pamphlets were supplied to the employees?

About which product did Brittany wonder whether several commercials will be brought to the meeting?

About which program did Olivia find out whether several courses should be advertised to the engineers?

About which candidate did Daniel ask whether several editorials would be released to the press?

About which bike will several ads be shown to the athletes?

About which car would several articles be given to the executives?

About which teacher could several letters be sent to the kids?

About which castle would several books be awarded to the historians?

About which deodorant did Abigail find out how many commercials they will provide to the company?

About which author did Elizabeth ask how many courses they should propose to the provosts?

About which criminal do you wonder how many TV shows they could turn over to the police?

About which concert do you know how many videos they advertised to the distributors?

About which violinist did Jenny wonder whether they should distribute several documentaries to the theaters?

About which senator did Linda find out whether they would deliver several editorials to the staff?

About which saint did Ethan ask whether they could explain several sermons to the congregation?

About which drug did William wonder whether they delivered several pamphlets to the pharmacists?

About which region should they suggest several classes to the deans?

About which novel would they mail several lectures to the professors?

About which activities could they divulge several facts to the family?

About which phone did they show several websites to the purchaser?

Who did they display pictures of in the courtyard?

Who did they sell portraits of at the hotel?

Who did they show paintings of in the museum?

Who did they burn photographs of in the plaza?

Who were drawings of created at the factory?

Who were sketches of made at the university?

Who were films about produced in the studio?

Who were statues of built in the city?

Who were rumors about in the air at the school?

Who were models of in assembly in the workshop?

Who were sculptures of on exhibit in the gallery?

Who were reports of on the agenda in the meeting?

Did they hide remains of the victim in the house?

Did they conceal evidence of the prisoner in the basement?

Did they hear stories about the soldiers at the park?

Did they listen to lies about the defendant in the courtroom?

Were plays about the president seen at the theater?

Was footage of the burglar monitored in the store?

Was data about the immigrants examined at the station?

Was information about the students inspected at the center?

Were facts about the architect under study at the college?

Were images of the pope on sale at the market?

Were books about the artist for purchase at the exhibition?

Were documents about the spy in the trash at the library?
